# Evolutionary Constraints Improve Protein Large Language Model Predictions for Protein Stability, Binding Regions, and Epistasis

**DOI:** 10.34133/csbj.0226

**Published:** 2026-09-18

**Authors:** Konstantina Tzavella, Catharina Olsen, Wim Vranken

**Affiliations:** ^1^ Department of Medical Physics, Institut Jules Bordet, Hôpital Universitaire de Bruxelles (H.U.B), Université Libre de Bruxelles (ULB), Brussels 1070, Belgium.; ^2^ Interuniversity Institute of Bioinformatics (IB2), ULB-VUB, Brussels, Belgium.; ^3^ Brussels Interuniversity Genomics High Throughput Core (BRIGHTcore), Vrije Universiteit Brussel (VUB), Université Libre de Bruxelles (ULB), Brussels, Belgium.; ^4^ Clinical Sciences, Research Group Reproduction and Genetics, Centre for Medical Genetics, Vrije Universiteit Brussel (VUB), Universitair Ziekenhuis Brussel (UZ Brussel), Brussels 1090, Belgium.; ^5^ Structural Biology Brussels, Vrije Universiteit Brussel, Brussels, Belgium.; ^6^ Chemistry Department, Vrije Universiteit Brussel, Brussels, Belgium.; ^7^ AI Lab, Vrije Universiteit Brussel, Brussels 1050, Belgium.

## Abstract

Our understanding of protein function and evolution is largely based on the relationship between amino acid sequence and overall fold, now effectively captured by computational models. Yet, predicting how mutations—shaped by epistasis—alter protein behavior, especially in dynamic or structurally ambiguous regions, remains difficult. Here, we present D2D, which combines a self-supervised protein language model with protein-specific evolutionary information, to predict mutational effects using little to no task-specific labeled data. D2D captures long-range epistatic interactions and accurately predicts single and higher-order mutation effects on protein thermostability and binding, without being trained on the task. When fine-tuned, D2D outperforms state-of-the-art methods on latent driver cancer mutations and co-occurring proliferation-enhancing mutations across independent experimental studies. Unlike most existing approaches, D2D avoids biases linked to solvent accessibility or to multiple sequence alignment depth and quality, making it particularly effective for disordered or surface binding regions where structure-based predictors typically falter. Overall, D2D provides a general framework for modeling mutational effects in proteins with limited experimental or structural information.

## Introduction

Proteins are the molecular machines of all living systems, performing essential cellular functions from structural support and enzymatic activity to molecular transport. Their function is often determined and constrained by the 3-dimensional (3D) structure they adopt. In such a structure, amino acid residues interact so that changes at one site may require compensatory adjustments elsewhere to preserve packing, electrostatics, or flexibility [1–4]. These context-dependent mutational effects, known as intragenic epistasis, are pervasive in protein evolution [5–9], reverse genetic drift in small populations [9,10], and restore protein activity and influence drug resistance in cancer therapy [11–13]. For example, secondary mutations in BRCA2 can counteract primary mutations that are initially hypersensitive to chemotherapy drugs like cisplatin. By restoring the wild-type reading frame and protein structure of BRCA2, these secondary mutations ultimately drive the development of drug resistance.

Due to the complexity of proteins and amino acid interactions, quantitatively predicting or modeling the outcomes of intragenic epistasis remains difficult. The intricate behavior of proteins, the “protein phenotype”, is typically categorized by stability, which is highly relevant for well-folded protein domains such as enzymes, and functionality, which is typically closely related to conserved amino acids at functional sites or binding interfaces and motifs. Disrupting stability will, however, often also affect functionality, and separating these effects is not trivial, especially when multiple mutations are present.

Traditionally, multiple sequence alignments (MSAs), which capture evolutionary information (EI) on protein families, have been the established framework for inferring constraints on protein phenotype computationally [14–16]. Although MSAs provide explicit estimates of evolutionary constraints and residue coupling, their utility can be limited by alignment depth, family diversity, and computational demands, particularly when modeling higher-order dependencies such as interactions between multiple residues that mediate long-range allosteric effects. Nevertheless, most current computational predictors only evaluate the effect of single mutations or approximate multi-mutant outcomes under the assumption that their contributions are additive [17,18]. Recent methods have begun to address higher-order variants explicitly rather than assuming additivity, for example, by inferring the effects of high-order mutants from measured low-order variants [19]. However, such approaches typically still depend on experimental measurements for the protein of interest, leaving open how to capture epistatic effects when little or no such data are available. Moreover, the reliability of variant-effect predictors has been shown to depend on the structural context of the mutated position, with accuracy differing systematically between buried and solvent-exposed residues [20]. This positional bias is a recurring limitation of structure-informed approaches and motivates methods whose performance is stable across structural contexts.

Recently, protein language models (pLMs) have emerged as powerful tools in predicting tertiary structures [21], intrinsic disorder [22], single-cell annotations [23], and protein–protein interactions [24], even validating biological hypotheses [25]. Trained on massive protein sequence datasets, pLMs learn to model the statistical dependencies between amino acids based on their sequence context. Their alignment-free training produces rich, but extremely high-dimensional, embeddings, typically ~1,000 features per residue. This large embedding space poses a practical challenge for downstream models, particularly in data-limited settings, where it increases the risk of overfitting with many more features than samples. Moreover, unrealistic predictions of protein isoform structures have revealed that pLMs do not truly grasp the biophysics of proteins. Instead, they rely on statistical coevolutionary patterns, learned agnostically from many thousands of protein families [26].

More recent work has shown that incorporating EI into pLMs substantially improves stability-change prediction, with alignment-aware models outperforming single-sequence and structure-aware alternatives [27]. These gains, however, have largely been realized through supervised fine-tuning on large experimental stability datasets, which ties performance to the experimental conditions of the training data. It remains open whether the benefit of combining language-model and evolutionary signal can be obtained without task-specific training, and whether it extends to functional readouts such as binding in disordered regions. We further argue that this complementarity is most valuable in intrinsically disordered regions (IDRs), where sequence conservation is typically low and alignment-based methods lose discriminative power, yet where mutations can still carry substantial functional consequences. Because masked language modeling predicts residues from sequence context rather than from conservation alone, a substitution in a poorly conserved or flexible region can produce a large perturbation in embedding space, flagging a functionally relevant change that alignment statistics alone would miss.

Here, we address this with D2D, which combines the strengths of both: For each protein family, we constrain the general statistical representations learned by pLMs with protein-specific EI derived from MSAs. By modeling site-specific variation in embedding space using a Gaussian mixture model (GMM), we approximate the distribution of evolutionarily permissible states at each position. Mutational effects are then quantified as deviations from this distribution, enabling detection of substitutions that are statistically plausible in a global sense but incompatible with the local evolutionary constraints of the protein under study (Fig. 1). In this way, it requires little to no labeled data and remains applicable to disordered and surface regions where both structure-based and supervised approaches typically falter.

**Fig. 1.**
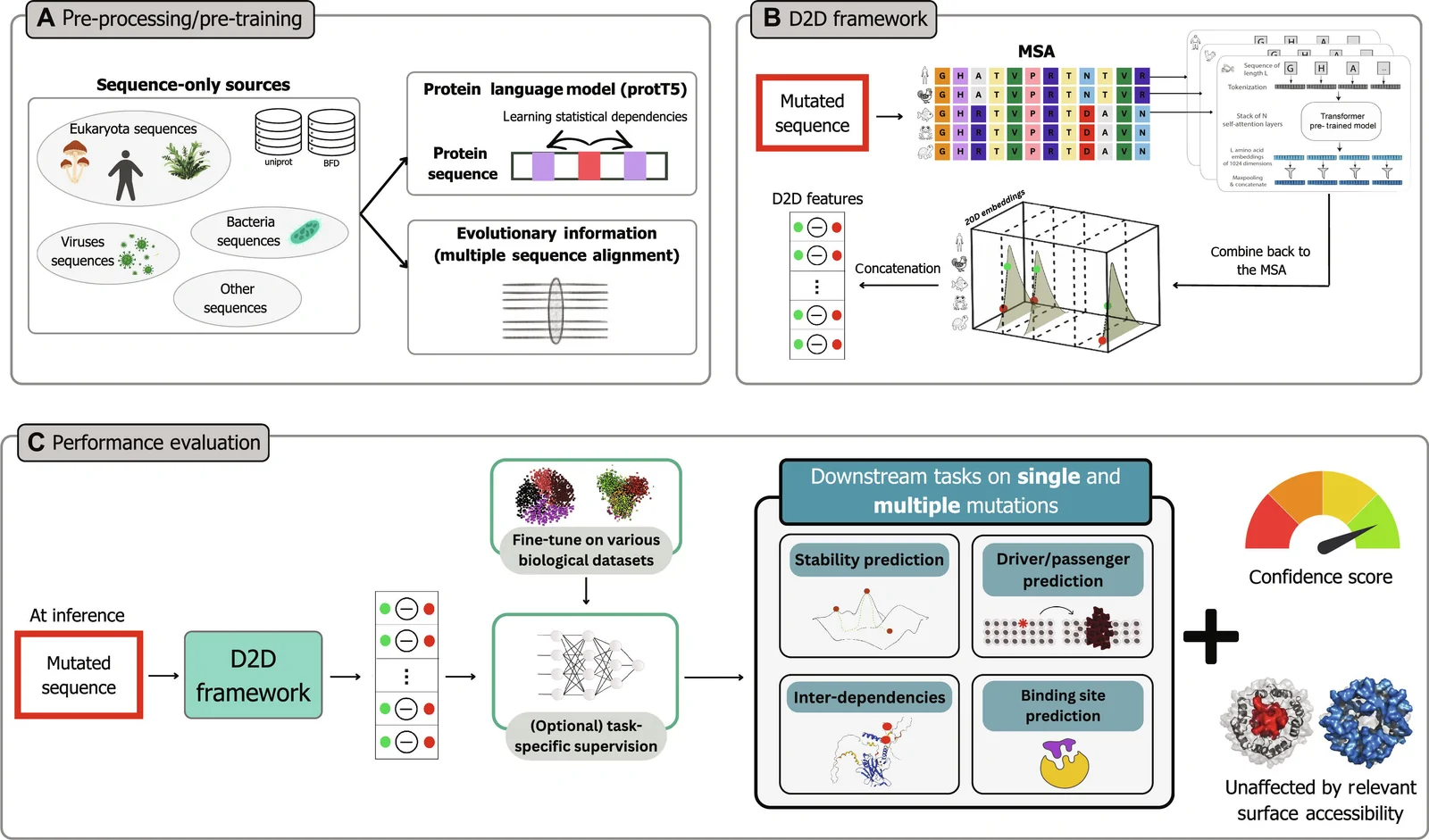
D2D pipeline. (A) A pLM pretrained on millions of protein sequences captures the statistical dependencies between amino acids. The MSA tool mmseqs2 uses the UniProt and BFD databases and captures the protein-specific distribution of the protein site. (B) Core D2D framework. Each amino acid of the MSA is mapped to a selected 20D pLM representation vector. The pLM vectors are combined back to the MSA, and a GMM is fitted at each position across species. The log-probability of the mutant representation is compared to that of the wild type, and the differences are concatenated to quantify mutational effects across the sequence. A 1D GMM is shown for illustration. (C) The resulting features can be used in an unsupervised manner (e.g., averaging) or as input to supervised models for downstream tasks. Outputs include prediction and confidence scores and enable identification of short- and long-range amino acid interactions and are minimally affected by relevant surface accessibility of the residue involved in mutation.

We have previously demonstrated the model’s effectiveness in predicting single cancer driver mutations [28] and now propose its general applicability to other biologically relevant tasks involving protein (mis-)behavior, such as protein stability prediction and binding site identification. This extended sequence-based framework (D2D) accurately identifies binding sites, including those located within IDRs, even under limited or no labeled data. Furthermore, it successfully detects novel, latent co-occurring mutations whose combined effects are associated with poorer treatment outcomes, capturing the nonlinear interactions that contribute to cancer progression.

## Results

To assess the broad effectiveness of combining pLM and EI for both single and multiple mutations, we focused on 3 key protein phenotypes: (a) protein stability and fitness, (b) binding site prediction with emphasis on linear interacting peptides (LIPs) that mediate protein–protein interactions, and (c) the impact of secondary, synergistic mutations on tumor growth.

### D2D demonstrates strong performance in protein stability and fitness prediction across solvent accessibility levels

Amino acid mutations that alter the thermodynamic stability of proteins are associated with various diseases. In cancer, for example, such mutations can cause tumor suppressor proteins to unfold, or can conversely stabilize oncogenic proteins. To evaluate the performance of our model on thermodynamic stability data, we used the Mega-scale dataset [29], which quantifies the effects of single amino acid substitutions on the free energy difference between folded and unfolded states (ΔΔ*G*). We benchmarked on 291,776 mutations, including 162,725 single and 129,051 double mutations, across 166 domains.

We benchmarked D2D against state-of-the-art (SOTA) predictors GEMME [30], mCSM [31], MSA_Transformer [32], and ESM-1v [17] that leverage evolutionary, structural, pLM, and evolutionary/pLM information, respectively (Fig. 2A). The unsupervised D2D approach, which is agnostic of thermodynamic stability information, already achieves Spearman correlation scores comparable to SOTA methods, significantly outperforming the well-established, specifically trained mCSM predictor (Wilcoxon test *P* = 0.003) and MSA_Transformer (*P* = 0). Because it captures the statistics of the variation of pLM embeddings, D2D can provide a confidence score for each prediction based on the quality of the Gaussian fit to the underlying MSA data. When restricting the analysis to the 24 studies with average confidence scores of at least 60%, all predictors showed improved performance. Notably, D2D’s Spearman correlation increased to 0.59, outperforming all other predictors (Fig. 2A). Confidence scores above 70% were excluded from further analysis due to insufficient data points.

**Fig. 2.**
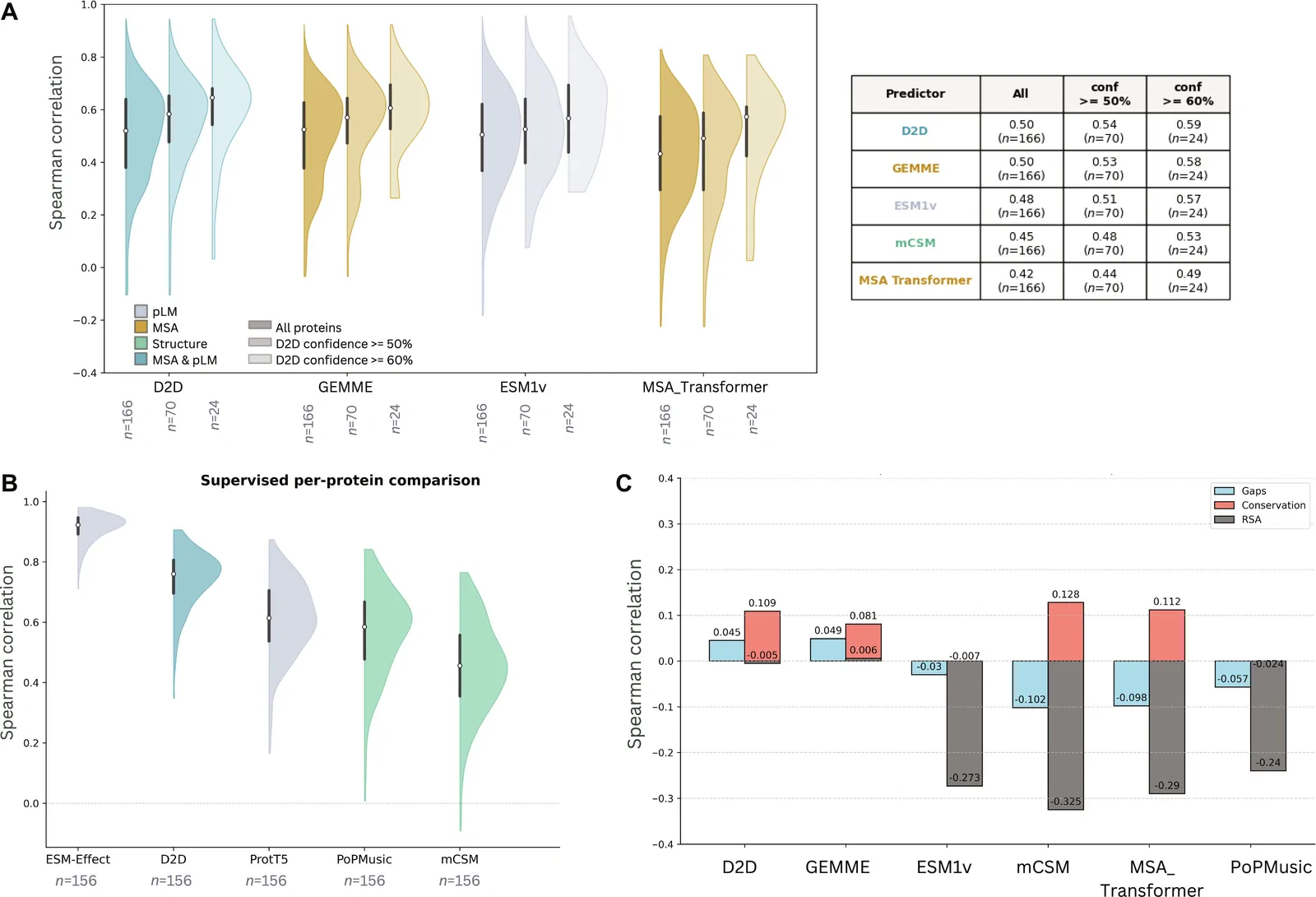
(A) D2D predictions compared to SOTA predictions on the mega-scale dataset of experimental ∆∆*G* values upon single and double mutations. All predictions, together with the subset scoring above 50% and 60% confidence, are shown in the same color at decreasing intensities. The table on the right summarizes each predictor’s performance. (B) Comparative performance of supervised predictors. ESM-Effect, D2D, and ProtT5 were fine-tuned as described in Materials and Methods. (C) Spearman correlations between the performance across 166 mega-scale studies and the corresponding average gaps, conservation, and RSA values per study. A negative Spearman correlation (e.g., for RSA) indicates that predictors perform worse for exposed residues (i.e., residues with high RSA).

Under an in-protein cross-validation setting, where the training and test sets share protein domains but contain disjoint mutations, the fine-tuned ESM-Effect model [33] substantially outperformed D2D followed by a multi-layer perceptron (MLP), as well as PopMusic, mCSM, and ProtT5 features followed by an MLP (Fig. 2B). This performance gap may be attributed to differences in the depth of supervision. In D2D + MLP, only the MLP head is trained, while the features are fixed and therefore cannot adapt to the target task. In contrast, ESM-Effect propagates the supervision signal through the transformer with ~6 million parameters, allowing the learned representation to adapt during fine-tuning, while its prediction head explicitly conditions on the mutated position. This distinction is particularly relevant under the in-protein split: With near-saturation mutagenesis, held-out substitutions at a given position are strongly constrained by other observed substitutions within the same protein. Consequently, part of the performance advantage may arise from exploiting protein-specific patterns learned during training, rather than from signal that generalizes across proteins.

In agreement with the conclusions of [23], D2D features demonstrate superior performance compared to those derived from the ProtT5 pretrained model in a supervised setting with cross-validation (Fig. 2B).

A major challenge the stability predictors face is their inherent biases built into their design. For evolutionary predictors, poor-quality MSAs can reduce the reliability of the conservation score, potentially affecting the overall pathogenicity prediction. Structure-based predictors exhibit a different bias, tending to classify mutations buried [low relative solvent accessibility (RSA)] in the protein core as pathogenic [34]. To investigate these tendencies, we evaluated the dependence of each predictor on key parameters: the proportion of gaps at the mutation site in the MSA, the conservation level at the mutated position, and the residue’s RSA.

Consistent with previous observations [35,36], the performance of the 2 structure-based predictors mCSM and PoPMusic deteriorated with the increase of RSA (Fig. 2C). In other words, structure predictors struggle to correctly estimate the impact of mutations in exposed residues, as residues are not packed together, making change in amino acid side chains more difficult to assess. Interestingly, the sequence-based pLM predictors MSA_Transformer and ESM-1v also appeared to be impacted by the RSA of the residue. In contrast, evolutionary models D2D and GEMME remain robust across varying RSA levels. This discrepancy may stem from 2 factors: (a) Structure-based methods often rely on static structural representations that inadequately capture the dynamic nature of exposed regions, and (b) mutations at exposed sites may primarily impact functional properties, such as binding or catalysis, rather than stability, which most ∆∆*G* predictors are designed to assess. Regarding the MSA conservation, we see a minimal impact on the performance, especially for mCSM, with almost all predictors performing better when conservation is higher. The presence of gaps in the MSA does not have any effect.

We extended our analysis to the fitness-prediction task from the ProteinGym database [6], benchmarking SOTA predictors in the zero-shot, unsupervised setting. These methods integrate structural, evolutionary, and pLM-derived information, and most performed comparably (Fig. 3A). A subsequent RSA analysis showed that, among the top-performing methods, D2D was the least sensitive to RSA value and MSA quality (Fig. 3B).

**Fig. 3.**
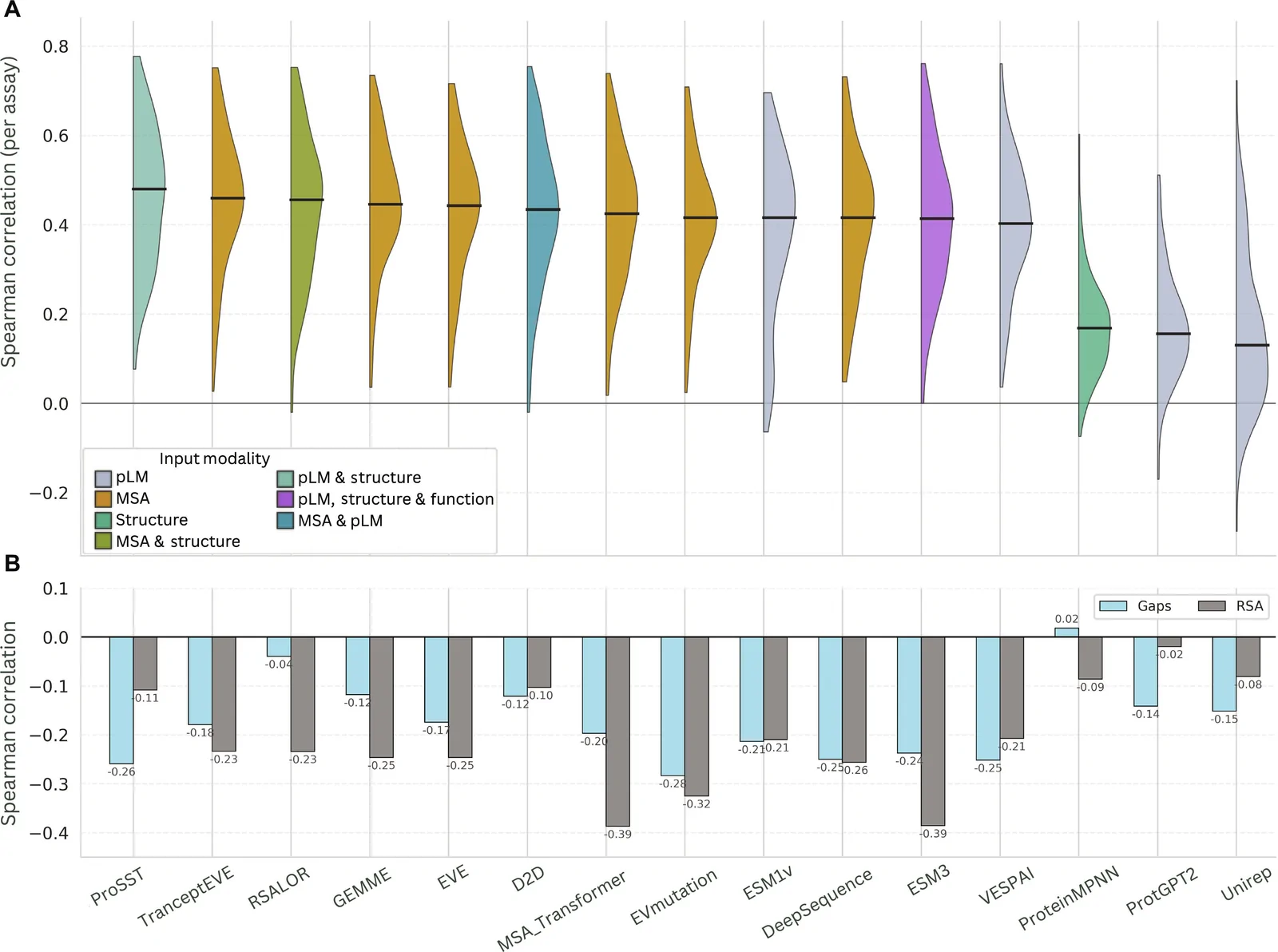
(A) Comparison of SOTA predictors on the ProteinGym dataset. (B) Spearman correlations between the performance across 126 studies and the corresponding average gaps and RSA values per study. A negative Spearman correlation (e.g., for RSA) indicates that predictors perform worse for exposed residues (i.e., residues with high RSA).

### Compensatory mutation detection

We have previously shown that D2D features capture distal mutational effects, consistent with other structure-based predictors, and, additionally, correlate with residues that are spatially proximal [28]. To investigate D2D’s ability to deal with multiple mutations, we focused on the compensatory mutations, where the negative effects of one mutation are alleviated by epistatic interaction with a second mutation at another site. Although not fully understood, this phenomenon plays a critical role in important biological processes like antibiotic resistance [37] and cancer therapy [12]. In tumors, for instance, mutations in sites targeted by drugs can directly or indirectly interfere with their binding, leading to drug resistance [13].

We hypothesized that by constraining the protein-agnostic coevolutionary statistics with protein-specific EI, we can uncover relationships between mutations and protein sites that are prone to interact. To do so, we first defined sites as epistatic when their D2D signal was greater than 10% of the global maximum D2D signal across all sites. From the mega-scale dataset, we then selected single mutations that are destabilizing (ΔΔ*G* < −2) but become stabilizing (ΔΔ*G* > 1) upon introduction of a second mutation, following the stabilizing threshold proposed in [24]. The double stable mutants on epistatic sites were 868 compared to 623 on non-epistatic sites. In contrast, destabilizing mutations were more frequently observed at non-epistatic sites than at epistatic ones (Fig. 4A). Overall, double mutations that restore stability are enriched 1.4-fold at epistatic sites relative to non-epistatic sites (odds ratio ≈ 1.43, *P* ≈ 6 × 10^−12^, Fisher test), supporting our hypothesis.

**Fig. 4.**
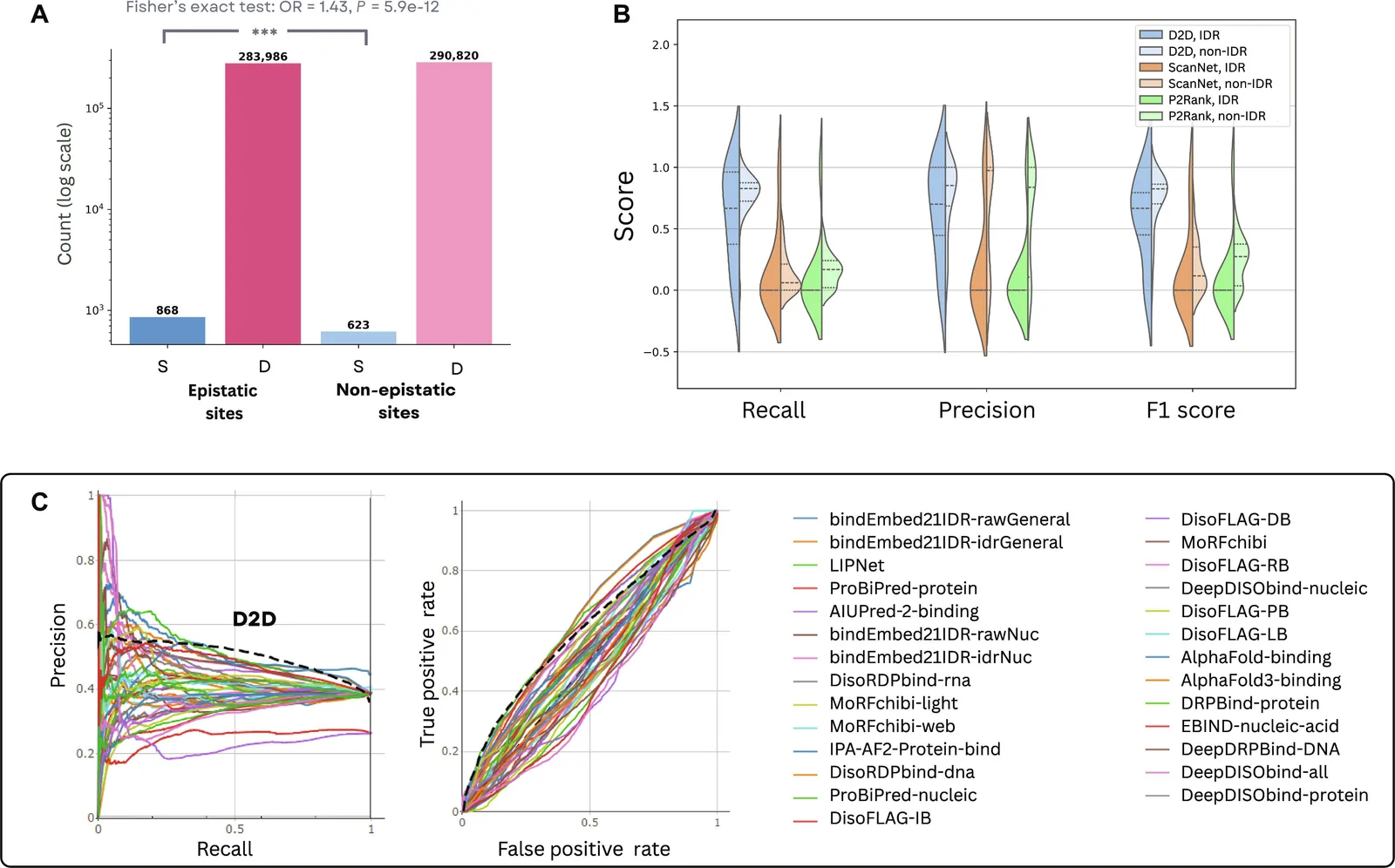
(A) Epistatic sites were defined as positions where the D2D signal exceeded ^1^/_10_ of its maximum value. Compensatory mutations (secondary mutations that alleviate the negative effects of a single mutation) occur more frequently (odds ratio ≈ 1.43, *P* ≈ 6 × 10^−12^, Fisher test) on epistatic sites than on non-epistatic sites. (B) Comparison of D2D-derived LIP predictions with ScanNet and P2Rank in IDRs and ordered regions. (C) Evaluation on CAID-3 Challenge binding-ID benchmark, Precision and Recall, and true positive rate (TPR)–false positive rate (FPR) curves.

### Binding site prediction in ordered and disordered regions

LIPs are functional motifs involved in binding and intermolecular interactions, mostly found in IDRs. In these poorly structured regions, sequence conservation is typically low, making it difficult to assess the impact of mutations, even though their functional impact can be high. We therefore sought to evaluate whether the combination of pLMs and EI features works in this challenging case.

We compared LIPs from the mega-scale collection, annotated in the MobiDB database [38], with interaction sites predicted using our approach as described in Materials and Methods. These results were benchmarked against 2 SOTA predictors: P2Rank [39] and ScanNet [40]. P2Rank is a machine learning method trained to predict ligand binding sites from protein structures. ScanNet is a structure-based predictor that uses geometric deep learning to learn spatio-chemical patterns of binding sites directly from 3D structures, also specifically trained to predict binding sites.

We hypothesized that mutations with minimal impact on protein stability but strong evolutionary constraint are enriched in functional sites. To investigate this, we combined experimental stability measurements (∆∆*G*) with evolutionary scores to construct mutational landscapes capturing low stability impact and high evolutionary effect. We evaluated performance separately for IDRs and ordered regions. As summarized in Fig. 4B, our method, D2D, outperforms both P2Rank and ScanNet for binding sites in both IDRs and ordered regions, although all predictors perform better on ordered regions compared to IDRs.

To further validate our approach, we tested D2D on the CAID-3 Challenge binding-IDR dataset [40], comprising 52 sequences, and compared its performance to the other algorithms that participated in the challenge (Fig. 4C), confirming its superior predictive ability in disordered regions. The CAID evaluation framework currently permits only central processing unit (CPU)-based execution under constrained computational resources. As discussed in the Computational requirements section, these limitations prevent on-the-fly benchmarking of D2D within the CAID competition due to the graphics processing unit (GPU) requirements associated with ProtT5 inference.

### Latent driver mutation discovery and their effect on tumor growth

As the D2D approach is relevant for single amino acid driver versus passenger classification in cancer [28], we further explored its performance on genes where double mutations increase oncogenic activity. Findings in [41] indicate that combining frequent driver mutations with rare or weaker, latent, mutations in the same gene can enhance tumor progression and affect treatment outcomes. To investigate this, we used the mutations reported in that study and created a negative (control) set from gnomAD [42] for the same genes, as described in Materials and Methods. In total, 25,992 pathogenic and 22,743 benign mutations across 18 genes were tested. We next benchmarked the cancer-specific version of D2D, D2Deep (described in Materials and Methods), against 4 established predictors: AlphaMissense [43], GEMME, EVE [44], and FATHMM-cancer [45]. Across the 18 genes for which all predictors could be evaluated, D2Deep performed comparably to AlphaMissense, achieving a higher mean F1 score (0.53 versus 0.50) but a slightly lower median F1 score (0.55 versus 0.57) (Fig. 5A). By comparison, GEMME, EVE, and FATHMM-cancer showed substantially lower performance, with median F1 scores of 0.25 across 18 genes, 0.01 across 8 genes, and 0 across 20 genes, respectively. AlphaMissense is based on AlphaFold-predicted protein structures and relies on trained model weights that are not publicly available. Thus, although its predictions provide a useful benchmark, the publicly available implementation cannot be independently used to generate new predictions. The results demonstrate that D2Deep achieves performance comparable to AlphaMissense while providing a fully accessible framework for generating cancer-specific mutation effect predictions.

**Fig. 5.**
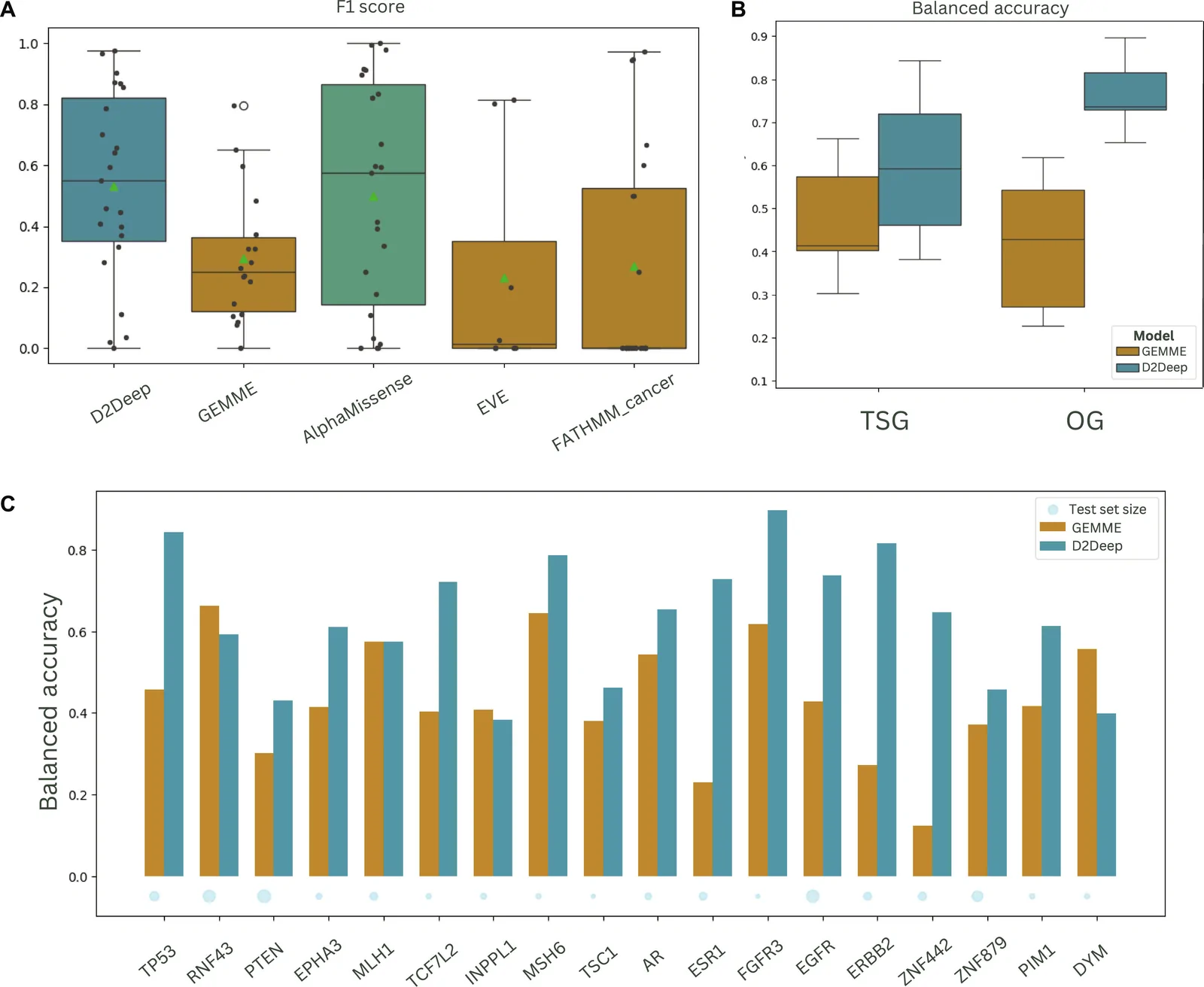
(A) F1 score for D2Deep, GEMME, AlphaMissense, EVE, and FATHMM_cancer predictors for latent mutations [42] and gnomAD control mutations. (B) D2Deep demonstrates better results for OGs compared to TSG. (C) Detailed performance of latent mutations and control mutations on 18 genes in relation with their test size.

From Fig. 5B, we also conclude that D2D performs better for oncogenes (OG) compared to tumor suppressor gene (TSG). This indicates that D2D is more sensitive in identifying the activation of OG (through gain-of-function mutations) than the inactivation of TSG (loss-of-function mutations), both of which are key processes in cancer development. Consistently across all evaluated genes, D2D outperforms GEMME, as further illustrated in Fig. 5C.

We then focused on driver mutation pairs of the well-known TP53 gene, by comparing the simple addition of individual predictions with the epistatic dual mutant prediction, across all suggested epistatic positions in [41] and for all amino acid combinations. The rationale underlying this analysis is that co-occurring mutations within the same gene may exhibit additive or cooperative effects. When the predicted effect of a double mutant equals the sum of the individual mutation effects, this indicates a lack of functional interaction. In contrast, when the phenotype of a double mutation cannot be explained by the additive effects of the individual single mutants, this indicates that epistatic links are present. When the combined mutations produce a phenotype that is more detrimental than expected, the interaction is classified as negative epistasis. In contrast, if the double mutant displays a milder phenotype than predicted, the interaction is considered positive.

To test whether D2Deep’s latent representation encodes epistatic interactions, we compared its double-mutant predictions at proposed epistatic (“latent-driver”) position pairs in TP53 against distance-matched, non-epistatic control pairs (Materials and Methods). Across all variants, the pre-sigmoid double-mutant score scaled approximately linearly with the sum of the 2 single-mutant scores but fell systematically below strict additivity (Fig. 6A), indicating that the predictor is intrinsically sub-additive—combining 2 mutations yields less than the sum of their individual effects. Because this saturation applies to epistatic and control pairs alike, we defined the empirical additive baseline as the linear trend through the control double mutants and quantified epistasis as each variant’s signed residual from this control trend, where a positive residual denotes a super-additive response than a non-epistatic pair of equivalent single-mutant magnitude. Under this baseline, validated latent driver double mutants from [41] (“O” marks in Fig. 6A) departed upward from the control trend significantly more than their distance-matched controls, exceeding their matched control in 10 of 12 cases (paired Wilcoxon signed-rank test, *P* = 0.002; Fig. 6B). Consistent with this, absolute departures from additivity were roughly 2-fold greater for epistatic than for control double mutants (Fig. 6C). To determine whether the signal was specific to the validated substitutions or a broader property of the position pairs, we examined the full saturation-mutagenesis distribution at each of the 4 pairs containing validated drivers (Fig. 6D). Validated substitutions fell predominantly in the super-additive region of their pair’s distribution, exceeding the pair median in 10 of 12 cases (mean within-pair percentile = 71.1%; one-sided Wilcoxon *P* = 0). At the same time, 3 of the 4 pairs showed that the entire saturation cloud shifted above the control baseline (median residual = +4.85, +2.40, and +1.43 logit units for 285:271, 160:161, and 285:287, respectively; 280:287 was marginally below at −1.28). Together, these indicate that the proposed epistatic positions are broadly coupled at the site level, with the experimentally validated substitutions representing the more strongly super-additive members of each pair, a distinction between coupled sites and coupled substitutions that D2Deep captures at both levels.

**Fig. 6.**
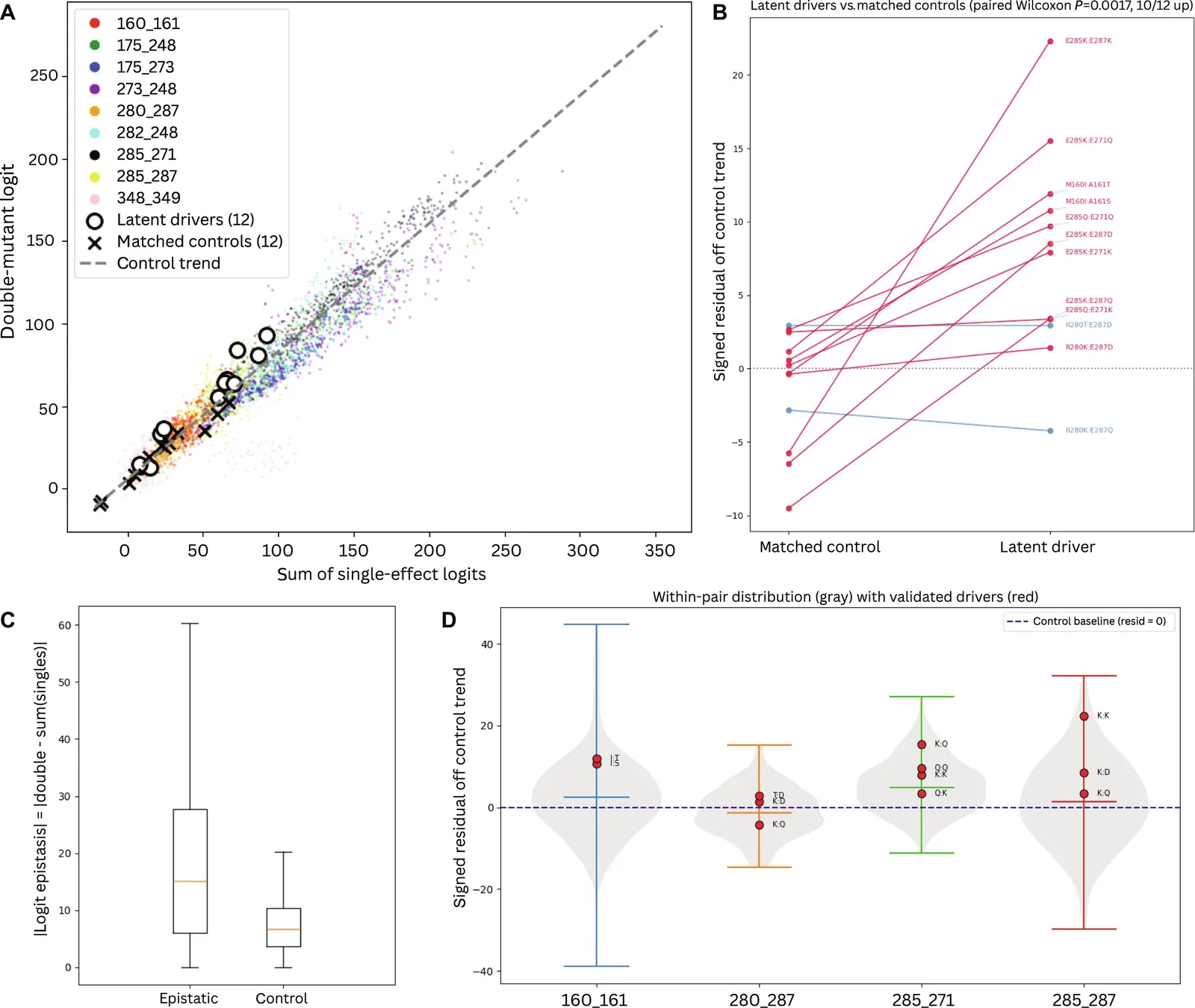
D2Deep predictions are more super-additive at validated TP53 latent-driver pairs than at matched controls. (A) Pre-sigmoid double-mutant scores (logits) versus the sum of the 2 single-mutant logits, for all saturation double mutants at the 9 proposed epistatic position pairs (colored by pair) and at 9 distance-matched non-epistatic control pairs. Dashed line, linear control trend (fit to control doubles), i.e., the model’s expected additive relationship in the absence of coupling. “O” marks are the 12 experimentally validated latent driver double mutants; crosses, their distance-matched controls. (B) Signed residual from the control trend for each validated latent driver and its distance-matched control (paired). Drivers departed above the baseline more than their matched control in 10/12 pairs (one-sided paired Wilcoxon signed-rank test, *P* = 0.0017); red, driver > control; blue, driver < control; labels denote the substitution pair. (C) Absolute departure from additivity (|epistasis| = |double − Σ singles|, logit units) for all epistatic (*n* = 3,249) versus control (*n* = 3,249) double mutants; epistatic variants deviate ≈2-fold more. (D) Distribution of signed residuals across the complete 19 × 19 saturation cloud (gray violins) at the 4 position pairs containing validated drivers; navy dashed line, control baseline; red points, validated drivers. Validated substitutions lie predominantly in the super-additive (upper) region of their pair’s distribution (mean within-pair percentile = 71.1%; one-sided Wilcoxon *P* = 0).

Finally, to further illustrate the epistatic signal captured by D2D and its relevance on actionable clinical analysis, we selected PIK3CA, the most frequently mutated OG in human cancers. It has hotspot single-amino acid mutations commonly occurring in the helical domain (E542K, E545K) and kinase domain (H1047R). Clinical trials [46,47] have shown that some patients with particular double PIK3CA mutations experience prolonged benefit from alpelisib, suggesting that such double mutations may serve as a biomarker for enhanced response to phosphatidylinositol 3-kinase (PI3K) inhibitors. This prompted researchers to conduct an in-depth analysis of the prevalence and biological significance of these mutations in relation to PI3Kα inhibitor sensitivity. In [48], a growth proliferation time course study of PIK3CA mutant MCF10A cells, serum-starved over 4 d, revealed that cells with double mutations displayed increased proliferation compared to single mutants. We compared these experimental findings with the predictions from D2Deep, GEMME, and FATHMM-cancer models. D2Deep accurately predicted the increase of mutation effect in correct order for 7 of 10 mutation effects, outperforming AlphaMissense (5 correct predictions), EVE and GEMME (4 correct), and FATHMM-cancer (3 correct predictions) (Fig. 7).

**Fig. 7.**
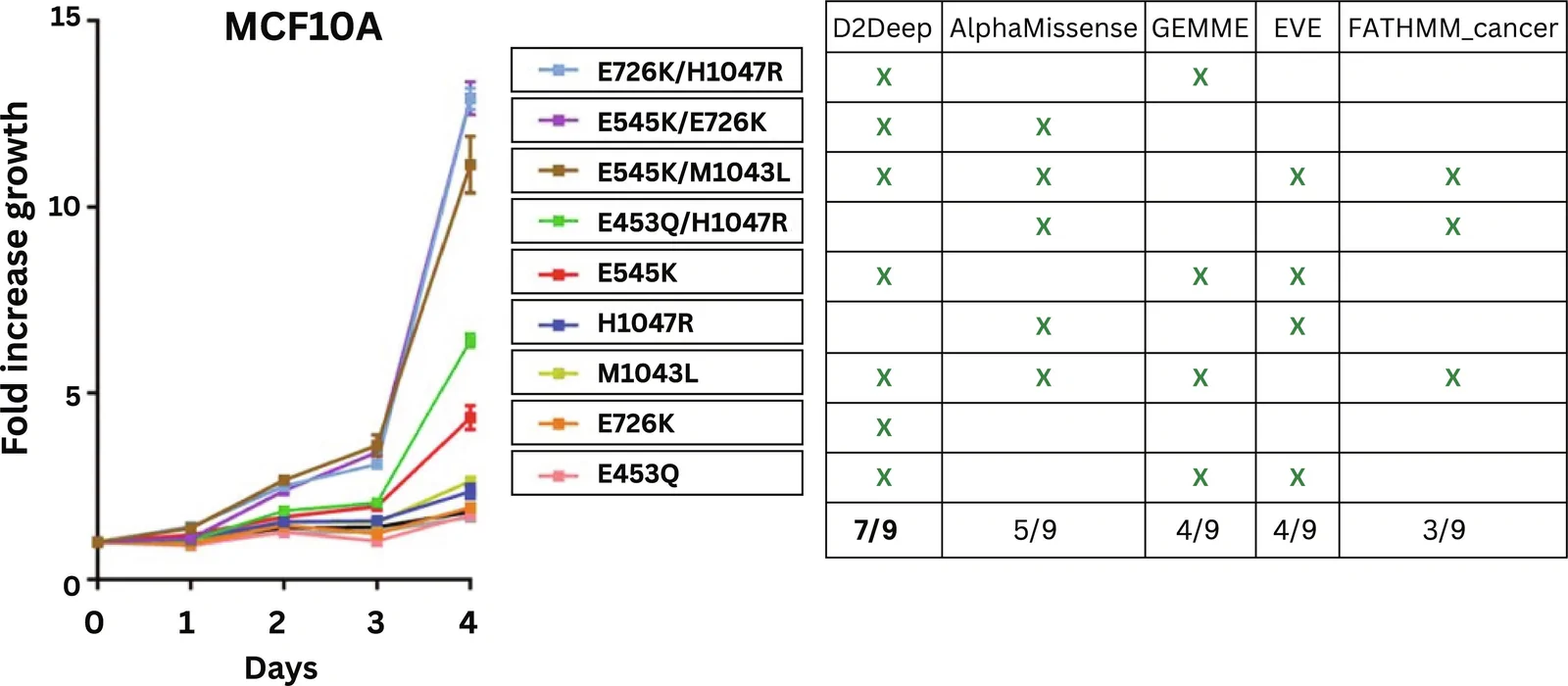
D2Deep, GEMME, AlphaMissense, EVE, and FATHMM-cancer mutation effect prediction for PIK3CA single and double mutants.

## Discussion

pLMs have shown remarkable potential for addressing diverse problems in computational biology. However, their general-purpose representations can struggle to capture specialized biological knowledge that depends on specific evolutionary or structural contexts. D2D addresses this limitation by constraining the pLM-derived embeddings with explicit EI obtained from sequence alignments.

Here, we present a proof of concept of this approach by evaluating the predictive capability of D2D across several biological tasks, including protein stability and binding. In the context of stability prediction, D2D shows reduced bias with respect to residue accessibility compared with many current SOTA approaches. In particular, the model is able to capture stability effects arising from mutations located on the protein surface that are epistatically coupled with residues in the structural core. This suggests that combining pLM representations with evolutionary constraints can help capture long-range dependencies that influence stability.

Interestingly, D2D also performs well on binding-related tasks despite not being explicitly trained on protein–protein interaction data. We interpret this as evidence that the relative contribution of the pLM and EI components shifts with the task. In stability prediction, columns of the MSA carry strong signal because structurally important residues tend to be highly conserved. In binding, however, residues that are critical for interaction but do not strongly affect stability can remain weakly conserved, limiting the information available from MSA-derived statistics alone. Here, the pLM component appears to provide complementary information: Because masked language modeling predicts residues from sequence context, substitutions—even in flexible or poorly conserved regions—can produce large embedding perturbations that flag a functionally relevant change.

This finding suggests that models such as D2D could potentially be extended to large-scale analyses of intergenic epistasis and protein–protein interaction prediction. To achieve this, future extensions would need to incorporate representations capable of modeling interchain dependencies. One promising direction would involve replacing the current sequence embeddings with complex-aware representations derived from models trained on protein–protein complexes. In such a framework, the current approach of fitting one GMM per residue position based solely on a protein’s MSA could be generalized. Instead, GMMs could be trained on embeddings representing co-evolved residue pairs or conserved interface motifs. D2D could then compute 2 complementary quantities: intra-chain distances, measuring how well a mutant residue fits within the evolutionary distribution of its own protein, and interchain distances, reflecting how compatible that mutation is with the interface features of its binding partner.

Another strength of D2D is its robustness in data-limited settings. The model performs well even with weak supervision and small labeled datasets. Beyond single mutations, applying D2D to co-occurring variants provides a potential framework for analyzing mutational landscapes in cancer and may help identify functionally relevant mutation combinations for improved patient stratification.

Overall, our results demonstrate that refining pLM representations with evolutionary constraints within a probabilistic framework enables the analysis of mutational effects across diverse biological contexts with minimal supervision. Future work incorporating complex-aware representations and explicit modeling of co-evolving interfaces may further extend this approach to predicting interaction-specific epistasis and large-scale protein–protein interaction networks.

## Materials and Methods

### D2D architecture and extension

This work builds on the method introduced in [28], in which a supervised D2D framework was developed for cancer driver mutation prediction. Briefly, for each mutated protein sequence, a genetic database search is performed to construct an MSA across diverse species. Each amino acid in the MSA is then mapped to a 20D pLM representation vector, which is selected from the available embeddings by max-pooling along the feature axis. We used a parameter-free operator by design: Learned alternatives such as principal components analysis (PCA) require fitting, which in the low-data, family-specific D2D targets risks overfitting and leaks information into the per-protein evaluation. Max-pooling is preferred over mean-pooling because ProtT5 embeddings are sparse with high-magnitude outlier dimensions that averaging would dilute, because the signed coordinates make mean-pooling prone to sign cancellation within a block, and because max-pooling retains the high-variance coordinates where mutational perturbations are most pronounced while the trade-off is greater sensitivity to noise.

We reduced the feature dimensions using max-pooling with a kernel size of 50 and a stride of 50, resulting in 20D vectors for each amino acid. Given 20 × 50 = 1,000, dimensions beyond this range (i.e., the final 24) were excluded from the analysis. Retaining these samples would require zero-padding the signal to the next multiple of 50; however, we opted not to do so, as padding introduces artificial values that could bias the pooled maxima. Since the omitted portion represents less than 2.4% of the total input length, its impact on the resulting features is negligible.

The dimensionality is bounded above by the minimum MSA depth (24), because we intended to maximize the subsequent GMM log-likelihood while avoiding overfitting. We therefore selected the highest dimensionality maximizing GMM likelihood within this limit: Testing kernels of 205, 102, 50, and 25 (yielding 5, 10, 20, and 40 features), a kernel of 50 (20 dimensions) gave the best fit without loss of downstream performance (Table S3).

The 20D representations are integrated back into the MSA, and a GMM of component 1 is fitted to the representations to model the distribution at each alignment position across species. For a given mutation, the GMM log-probability of the mutant representation is subtracted from that of the corresponding wild-type representation (Fig. S3). The distributions of MSA depths are shown in Fig. S1. The resulting differences are concatenated across all sequence positions to capture the global effect of the mutation.

Unsupervised setting: The concatenated features are averaged.

Minimally supervised setting: A small MLP was used, with a hidden dimension of 16, batch of 32, a dropout rate of 0.1, and a LeakyReLU activation function. The network was trained for 10 epochs using AdamW optimizer with a learning rate of 0.01 and MSELoss function. Fivefold cross-validation was performed separately for each protein, ensuring that mutations in the training and test sets were nonoverlapping.

D2Deep cancer driver mutation predictor: The classifier is composed of 2 fully connected (FC) layers followed by a batch normalization for faster and more stable training. Because the FC layers must have a defined input length, we chose as maximum length the 2,200 amino acids, padding shorter sequences with zero. The model was trained for 200 epochs. The number of training epochs was selected using early stopping, whereby the chosen epoch corresponds to the point at which the validation error begins to increase while the training error continues to decrease. Dropout layers with a rate of 0.3 were applied to all layers during training to mitigate overfitting, each followed by a rectified linear unit (ReLU) activation function. The final FC layer determines pathogenicity via a sigmoid activation, which is incorporated within the BCEWithLogitsLoss loss function. The classifier weights were initialized using Xavier normal initialization, and the batch size was set to 64. Rather than relying solely on the current gradient at each update step, gradient descent with a momentum of 0.1 aggregates current and past gradients. The optimizer was AdamW, with a learning rate of 3 × 10^−5^ and a scheduler employing a warmup period of 1,000 steps. All models were implemented and trained in the PyTorch framework.

### Thermodynamic protein stability dataset

We leveraged a mega-scale dataset [29] that quantifies changes in thermodynamic stability (∆∆*G*) between folded and unfolded states upon the introduction of single and double substitutions under standardized conditions. The dataset was generated using a high-throughput cDNA proteolysis assay in a cell-free *in vitro* translation system derived from *Escherichia coli* and comprises 331 natural protein domains and 148 designed proteins, each up to 72 amino acids in length, across a diverse set of organisms, measured under identical conditions.

To ensure data reliability, we excluded the domains with low-confidence ∆∆*G* measurements, as well as mutated structures that could not be mapped to a UniProt reference sequence. This resulted in 291,776 mutations, including 162,725 single and 129,051 double mutations, across 166 domains. Following the definition used by Sledzieski *et al*. [24], mutations with ∆∆*G* > 1 kcal/mol were classified as stabilizing. For this dataset, D2D predictions were generated in an unsupervised manner by averaging the corresponding D2D feature representations.

### Effect of mutations on protein fitness leveraging ProteinGym database

ProteinGym [18] provides a comprehensive collection of 250 deep mutational scanning (DMS) experiments, covering a wide range of mutations (both substitutions and indels) along with phenotypic measurements that are known or hypothesized to be associated with protein fitness. Of these studies, 148 focus on single and multiple substitutions across diverse functional properties, including ligand binding, aggregation, thermostability, viral replication, and drug resistance. In total, the dataset contains nearly 300,000 mutations, spanning a broad spectrum of protein families—such as kinases, ion channel proteins, G protein-coupled receptors, polymerases, transcription factors, and tumor suppressors—and covering proteins from multiple taxa. Even though DMS provide valuable data for studying mutation effects, several limitations constrain their use as benchmarks. Firstly, measurement noise and restricted dynamic ranges introduce variability among studies (Fig. S5). Selection bias arises from experimental feasibility and prioritization of certain proteins, such as viral or cancer-related proteins. Limited representativeness stems from assays often targeting a single molecular property rather than overall organismal fitness. Finally, heterogeneous data processing across studies can lead to inconsistent reported effects. Together, these factors necessitate careful interpretation of DMS-based evaluations.

### MobiDB LIPs sites and CAID-3 Challenge

Functional sites were annotated using MobiDB, a database that compiles disorder annotations for 245.5 million proteins, integrating experimentally validated and curated information on IDRs and LIPs. Protein domains from the mega-scale dataset were mapped to their corresponding UniProt sequences, accounting for cases where experimental constructs included truncated or extended regions. Disorder and LIP annotations were then retrieved for the mapped regions. We retained mutations with small stability effects (−2 < ∆∆*G* < 1 kcal/mol) and low D2D scores (below the 60th percentile; Fig. S2). The D2D predictions were computed in an unsupervised way, by averaging the D2D features. For the functional annotation, we only considered high-confidence evidence from MobiDB, including experimentally supported annotations (curated and derived) and the highest-confidence disorder predictions, as detailed in Table S2.

### Latent mutation analysis

To define an appropriate negative control set, we leveraged population variation data from the Genome Aggregation Database (gnomAD) database (https://gnomad.broadinstitute.org/). For each gene of interest—identified as latent candidates in [41]—we retrieved common variants with allele frequency greater than 0.5%, under the assumption that such variants are more likely to be tolerated. We then generated double mutations combining these common variants within each gene, producing a set of putatively benign mutation pairs (gnomAD_latent_pairs). In total, 25,992 pathogenic and 22,743 benign mutations were tested in 18 genes that contained both latent driver pairs and corresponding gnomAD-derived variants (latent_driver_pairs). Predictions for this dataset were generated using the supervised D2Deep framework, as described in [28]. For the epistatic analysis, we used raw D2D prediction scores prior to sigmoid transformation. This choice preserves the full dynamic range of the model outputs, enabling direct comparison of absolute effect sizes between additive and epistatic predictions. For the TP53 analysis, the 4 validated epistatic pairs and their distance-matched controls are as follows: 285:271 (*d* = 14 amino acids) → control 193:207, 285:287 (*d* = 2 amino acids) → control 132:134, 160:161 (*d* = 1 amino acid) → control 311:312, 280:287 (*d* = 7 amino acids) → control 122:129.

### Computational requirements

The per-residue pLM embeddings and GMM fitting were performed on a TU102 GPU (GeForceRTX 2080Ti) equipped with 11 GB of GDDR6 memory. Due to the computational demands of ProtT5, our current implementation is restricted to input sequences no longer than 2,200 amino acids. As an alternative to on-the-fly embedding generation, one could use precomputed embeddings available from the UniProt database (approximately 396 GB required). Moreover, at the time of writing, embeddings are only available for reviewed sequences, while MSAs may also include unreviewed entries. To explore possible optimizations, we analyzed which components of the pipeline could be made more efficient. For a representative protein of 344 amino acids (approximately the median length of a human protein) with an MSA of 647 sequences, the total computation time for all possible single mutations is summarized in Table S4. From these results, we identified the mapping of ProtT5 embeddings across the 3D MSA array as the most time-consuming step. To mitigate this, we tested the time and storage requirements for saving and reloading the 3D embedding array (sequence length × MSA depth × embedding dimensions) for reuse in subsequent analyses. For instance, when evaluating alternative mutations or mutation combinations, it is sufficient to reload the MSA embeddings (with the wild-type sequence) and compute only the mutant embeddings. The framework requires Python 3.10 or later together with the following packages: PyTorch, Transformers, EVcouplings, scikit-learn, and Biopython. GPU acceleration is strongly recommended for ProtT5 inference.

## Data Availability

We provide the D2D features calculated for the mega-scale and CAID_binding_idr datasets, the latent_driver_pairs, and the gnomAD_latent_pairs in the Zenodo repository: https://doi.org/10.5281/zenodo.20309165. The D2D code, together with instructions for reproducing the results and the associated software and hardware requirements, is available at: https://github.com/KonstantinaT/D2D.

## Supplementary Materials

Supplementary 1 Figs. S1 to S5 Tables S1 to S3 Reference [49]

## References

[1] Peccati F, Segovia CM, Núñez-Franco R, Jiménez-Osés G. Computation of protein thermostability and epistasis. Wiley Interdiscip Rev Comput Mol Sci. 2025;15(5): Article e70045.

[2] National Library of Medicine (US), National Institutes of Health. Protein folding and the thermodynamic hypothesis, 1950–1962. Christian B. Anfinsen—Profiles in Science. 2019.

[3] Surpeta B, Sequeiros-Borja CE, Brezovsky J. Dynamics, a powerful component of current and future in silico approaches for protein design and engineering. Int J Mol Sci. 2020;21(8):2713. 10.3390/ijms21082713 PMC7215530 32295283

[4] Dishman AF, Volkman BF. Design and discovery of metamorphic proteins. Curr Opin Struct Biol. 2022;74: Article 102380. 10.1016/j.sbi.2022.102380 PMC9664977 35561475

[5] Domingo J, Diss G, Lehner B. Pairwise and higher order genetic interactions during the evolution of a tRNA. Nature. 2018;558(7708):117–121. 10.1038/s41586-018-0170-7 PMC6193533 29849145

[6] Gonzalez CE, Ostermeier M. Pervasive pairwise intragenic epistasis among sequential mutations in TEM-1 β-lactamase. J Mol Biol. 2019;431(10):1981–1992. 10.1016/j.jmb.2019.03.020 PMC6502654 30922874

[7] Lipsh-Sokolik R, Fleishman SJ. Addressing epistasis in the design of protein function. Proc Natl Acad Sci USA. 2024;121(34): Article e2314999121. 10.1073/pnas.2314999121 PMC11348311 39133844

[8] Breen MS, Kemena C, Vlasov PK, Notredame C, Kondrashov FA. Epistasis as the primary factor in molecular evolution. Nature. 2012;490(7421):535–538. 10.1038/nature11510 23064225

[9] Miton CM, Tokuriki N. How mutational epistasis impairs predictability in protein evolution and design. Protein Sci. 2016;25(7):1260–1272. 10.1002/pro.2876 PMC4918425 26757214

[10] Poon A, Otto SP. Compensating for our load of mutations: Freezing the meltdown of small populations. Evolution. 2000;54(5):1467–1479. 10.1111/j.0014-3820.2000.tb00693.x 11108576

[11] de Visser JAGM, Elena SF. The evolution of sex: Empirical insights into the roles of epistasis and drift. Nat Rev Genet. 2007;8(2):139–149. 10.1038/nrg1985 17230200

[12] Sakai W, Swisher EM, Karlan BY, Agarwal MK, Higgins J, Friedman C, Villegas E, Jacquemont C, Farrugia DJ, Couch FJ, et al. Secondary mutations as a mechanism of cisplatin resistance in BRCA2-mutated cancers. Nature. 2008;451(7182):1116–1120. 10.1038/nature06633 PMC2577037 18264087

[13] Kamaraj B, Bogaerts A. Structure and function of p53-DNA complexes with inactivation and rescue mutations: A molecular dynamics simulation study. PLOS ONE. 2015;10(8): Article e0134638. 10.1371/journal.pone.0134638 PMC4526489 26244575

[14] Ng PC, Henikoff S. SIFT: Predicting amino acid changes that affect protein function. Nucleic Acids Res. 2003;31(13):3812–3814. 10.1093/nar/gkg509 PMC168916 12824425

[15] Adzhubei I, Jordan DM, Sunyaev SR. Predicting functional effect of human missense mutations using PolyPhen-2. Curr Protoc Hum Genet. 2013;76(1):20. 10.1002/0471142905.hg0720s76 PMC4480630 23315928

[16] Raimondi D, Tanyalcin I, Ferté J, Gazzo A, Orlando G, Lenaerts T, Rooman M, Vranken W. DEOGEN2: Prediction and interactive visualization of single amino acid variant deleteriousness in human proteins. Nucleic Acids Res. 2017;45(W1):W201–W206. 10.1093/nar/gkx390 PMC5570203 28498993

[17] Meier J, Rao R, Verkuil R, Liu J, Sercu T, Rives A. Language models enable zero-shot prediction of the effects of mutations on protein function. Adv Neural Inf Process Syst. 2021;34:29287–29303.

[18] Notin P, Kollasch A, Ritter D, Van Niekerk L, Paul S, Spinner H, Rollins N, Shaw A, Orenbuch R, Weitzman R, et al. ProteinGym: Large-scale benchmarks for protein fitness prediction and design. Adv Neural Inf Process Syst. 2023;36:64331–64379.

[19] Forrest B, Derbel H, Zhao Z, Liu Q. MMRT: MultiMut recursive tree for predicting functional effects of high-order protein variants from low-order variants. Comput Struct Biotechnol J. 2025;27:672–681. 10.1016/j.csbj.2025.02.012 PMC11894328 40070521

[20] van der Flier F, Estell D, Pricelius S, Dankmeyer L, van Stigt Thans S, Mulder H, Otsuka R, Goedegebuur F, Lammerts L, Staphorst D, et al. Enzyme structure correlates with variant effect predictability. Comput Struct Biotechnol J. 2024;23:3489–3497. 10.1016/j.csbj.2024.09.007 PMC11491678 39435338

[21] Weißenow K, Heinzinger M, Rost B. Protein language-model embeddings for fast, accurate, and alignment-free protein structure prediction. Structure. 2022;30(8):1169–1177. 10.1016/j.str.2022.05.001 35609601

[22] Ilzhöfer D, Heinzinger M, Rost B. SETH predicts nuances of residue disorder from protein embeddings. Front Bioinform. 2022;2:1019597. 10.3389/fbinf.2022.1019597 PMC9580958 36304335

[23] Cui H, Wang C, Maan H, Pang K, Luo F, Duan N, Wang B. scGPT: Toward building a foundation model for single-cell multi-omics using generative AI. Nat Methods. 2024;21(8):1470–1480. 10.1038/s41592-024-02201-0 38409223

[24] Sledzieski S, Kshirsagar M, Baek M, Dodhia R, Lavista Ferres J, Berger B. Democratizing protein language models with parameter-efficient fine-tuning. Proc Natl Acad Sci USA. 2024;121(26): Article e2405840121. 10.1073/pnas.2405840121 PMC11214071 38900798

[25] Rosen Y, Roohani Y, Agrawal A, Samotorčan L, Tabula Sapiens Consortium, Quake SR, Leskovec J. Universal cell embeddings: A foundation model for cell biology. bioRxiv. 2023 https://doi.org/10.1101/2023.11.28.568918. 10.1038/s41586-026-10689-z PMC13441871 42420460

[26] Zhang Z, Wayment-Steele HK, Brixi G, Wang H, Kern D, Ovchinnikov S. Protein language models learn evolutionary statistics of interacting sequence motifs. Proc Natl Acad Sci USA. 2024;121(45): Article e2406285121. 10.1073/pnas.2406285121 PMC11551344 39467119

[27] Cuturello F, Celoria M, Ansuini A, Cazzaniga A. Enhancing predictions of protein stability changes induced by single mutations using MSA-based language models. Bioinformatics. 2024;40(7):btae447. 10.1093/bioinformatics/btae447 PMC11269464 39012369

[28] Tzavella K, Diaz A, Olsen C, Vranken W. Combining evolution and protein language models for an interpretable cancer driver mutation prediction with D2Deep. Brief Bioinform. 2025;26(1): Article bbae664. 10.1093/bib/bbae664 PMC11663023 39708841

[29] Tsuboyama K, Dauparas J, Chen J, Laine E, Mohseni Behbahani Y, Weinstein JJ, Mangan NM, Ovchinnikov S, Rocklin GJ. Mega-scale experimental analysis of protein folding stability in biology and design. Nature. 2023;620(7973):434–444. 10.1038/s41586-023-06328-6 PMC10412457 37468638

[30] Laine E, Karami Y, Carbone A. GEMME: A simple and fast global epistatic model predicting mutational effects. Mol Biol Evol. 2019;36(11):2604–2619. 10.1093/molbev/msz179 PMC6805226 31406981

[31] Pires DE, Ascher DB. mCSM-AB: A web server for predicting antibody-antigen affinity changes upon mutation with graph-based signatures. Nucleic Acids Res. 2016;44(W1):W469–W473. 10.1093/nar/gkw458 PMC4987957 27216816

[32] Rao RM, Liu J, Verkuil R, Meier J, Canny J, Abbeel P, Sercu T, Rives A. MSA transformer. Int Conf Mach Learn. 2021;139:8844–8856.

[33] Glaser M, Brägelmann J. ESM-Effect: An effective and efficient fine-tuning framework towards accurate prediction of mutation’s functional effect. bioRxiv. 2025 https://doi.org/10.1101/2021.07.09.450648.

[34] Ramakrishnan G, Baakman C, Heijl S, Vroling B, van Horck R, Hiraki J, Xue LC, Huynen MA. Understanding structure-guided variant effect predictions using 3D convolutional neural networks. Front Mol Biosci. 2023;10:1204157. 10.3389/fmolb.2023.1204157 PMC10354367 37475887

[35] Pan Q, Nguyen TB, Ascher DB, Pires DEV. Systematic evaluation of computational tools to predict the effects of mutations on protein stability in the absence of experimental structures. Brief Bioinform. 2022;23(2): Article bbac025. 10.1093/bib/bbac025 PMC9155634 35189634

[36] Caldararu O, Mehra R, Blundell TL, Kepp KP. Systematic investigation of the data set dependency of protein stability predictors. J Chem Inf Model. 2020;60(10):4772–4784. 10.1021/acs.jcim.0c00591 32786698

[37] Piovesan D, Del Conte A, Clementel D, Monzon AM, Bevilacqua M, Aspromonte MC, Iserte JA, Orti FE, Marino-Buslje C, Tosatto SC. MobiDB: 10 years of intrinsically disordered proteins. Nucleic Acids Res. 2023;51(D1):D438–D444. 10.1093/nar/gkac1065 PMC9825420 36416266

[38] Tubiana J, Schneidman-Duhovny D, Wolfson HJ. ScanNet: An interpretable geometric deep learning model for structure-based protein binding site prediction. Nat Methods. 2022;19(6):730–739. 10.1038/s41592-022-01490-7 35637310

[39] Krivák R, Hoksza D. Improving protein-ligand binding site prediction accuracy by classification of inner pocket points using local features. J Cheminform. 2015;7(1):12. 10.1186/s13321-015-0059-5 PMC4414931 25932051

[40] Necci M, Piovesan D, Tosatto SC. Critical assessment of protein intrinsic disorder prediction. Nat Methods. 2021;18(5):472–481. 10.1038/s41592-021-01117-3 PMC8105172 33875885

[41] Yavuz BR, Tsai CJ, Nussinov R, Tuncbag N. Pan-cancer clinical impact of latent drivers from double mutations. Commun Biol. 2023;6(1):202. 10.1038/s42003-023-04519-5 PMC9941481 36808143

[42] Chen S, Francioli LC, Goodrich JK, Collins RL, Kanai M, Wang Q, Alföldi J, Watts NA, Vittal C, Gauthier LD, et al. A genomic mutational constraint map using variation in 76,156 human genomes. Nature. 2024;625(7993):92–100. 10.1038/s41586-023-06045-0 PMC11629659 38057664

[43] Tordai H, Torres O, Csepi M, Padanyi R, Lukacs GL, Hegedűs T. Analysis of AlphaMissense data in different protein groups and structural context. Sci Data. 2024;11(1):495. 10.1038/s41597-024-03327-8 PMC11094042 38744964

[44] Frazer J, Notin P, Dias M. Disease variant prediction with deep generative models of evolutionary data. Nature. 2021;599(7883):91–95. 10.1038/s41586-021-04043-8 34707284

[45] Shihab HA, Gough J, Cooper DN, Day IN, Gaunt TR. Predicting the functional consequences of cancer-associated amino acid substitutions. Bioinformatics. 2013;29(12):1504–1510. 10.1093/bioinformatics/btt182 PMC3673218 23620363

[46] Juric D, Rodon J, Tabernero J, Janku F, Burris HA, Schellens JH, Middleton MR, Berlin J, Schuler M, Gil-Martin M, et al. Phosphatidylinositol 3-kinase α-selective inhibition with alpelisib (BYL719) in PIK3CA-altered solid tumors: Results from the first-in-human study. J Clin Oncol. 2018;36(13):1291–1299. 10.1200/JCO.2017.72.7107 PMC5920739 29401002

[47] Juric D, Castel P, Griffith M, Griffith OL, Won HH, Ellis H, Ebbesen SH, Ainscough BJ, Ramu A, Iyer G, et al. Convergent loss of PTEN leads to clinical resistance to a PI(3)kα inhibitor. Nature. 2015;518(7538):240–244. 10.1038/nature13948 PMC4326538 25409150

[48] Vasan N, Razavi P, Johnson JL, Shao H, Shah H, Antoine A, Ladewig E, Gorelick A, Lin TY, Toska E, et al. Double PIK3CA mutations in cis increase oncogenicity and sensitivity to PI3Kα inhibitors. Science. 2019;366(6466):714–723. 10.1126/science.aaw9032 PMC7173400 31699932

[49] Hopf TA, Ingraham JB, Poelwijk FJ, Schärfe CP, Springer M, Sander C, Marks DS. Mutation effects predicted from sequence co-variation. Nat Biotechnol. 2017;35(2):128–135. 10.1038/nbt.3769 PMC5383098 28092658

