# Supplementary material for: Evolutionary Constraints Improve Protein Large Language Model Predictions for Protein Stability, Binding Regions, and Epistasis
Source: Comput Struct Biotechnol J. 2026 Sep 18;35(1):0226. doi: 10.34133/csbj.0226 (PMC13738275; doi:10.34133/csbj.0226)
Supplement: Supplementary file 1 [file csbj.0226.f1.docx]

**Supplementary Data**

**Supplementary Fig. 1:** MSA distributions for original and filtered MSA following the approach of Hopf et al [51]. The original MSA distributions have a minimum depth of 34 sequences, whereas the filtered MSAs have a reduced minimum depth of 24 sequences.

**Supplementary Fig. 2:** Pipeline for identifying and benchmarking LIPs using the D2D predictor. Protein structures are mapped to UniProt IDs, which are then used to retrieve functional annotations from MobiDB. For large-scale experimental datasets with available annotations, we select mutation sites that exhibit high conservation effects but low stability impacts. Predicted LIPs are compared against experimentally validated LIPs across both intrinsically disordered regions (IDRs) and structured (non-disordered) regions.

**Supplementary Fig. 3:**  The MSA with N sequences of length L is encoded into 1024-dimensional pLM embeddings. Max pooling reduces the embedding dimensionality to 20, after which each MSA column is used as a separate input to the multivariate GMMs. The MaxPooling1D layer applies a sliding window (kernel) of fixed size across the input sequence. For each window, the maximum value is selected, reducing the sequence length and retaining key features. The stride determines how far the window moves at each step—when stride equals the kernel size, the windows do not overlap.

**Supplementary Fig. 4:** Distributions of ΔΔG values for mega-scale mutations in IDR and non-IDR regions

**Supplementary Fig. 5: comparison of irregular distribution found in ProteinGym compared to examples of experimental values.**

**Supplementary Table 1:** Comprehensive overview of the performance of all predictors on the CAID-3 Binding-IDR dataset

**Supplementary Table 2:** MobiDB evidence level of selected annotations

**Supplementary Table 3:** Exploratory analysis using different max-pooling kernels to identify the optimal set of features that balances model performance and overfilling.
